# Reverse abdominoplasty: easily solving complicated situations

**DOI:** 10.1080/23320885.2025.2475902

**Published:** 2025-03-07

**Authors:** Daniele Brunelli, Francesca Mazzarella, Chiara Zanettin, Pasquale Zona, Diego Cappellina, Cesare Cappellina, Franco Bassetto, Vincenzo Vindigni

**Affiliations:** a Clinic of Plastic, Reconstructive and Aesthetic Surgery, Department of Neuroscience, University Hospital of Padua, Padua, Italy; b Clinic of Plastic Surgery, Cappellina Clinic, Dueville, Italy

**Keywords:** Reverse abdominoplasty, post-bariatric surgery, aesthetic surgery, body contouring

## Abstract

**Background:**

Epigastric tissue abundancy after abdominoplasty or liposuction is a complicated scenario that requires a precise and targeted approach. Especially when concurrent mammoplasty is planned or has already been done by the patient, a surgical operation through a submammary skin incision can solve this problem.

**Aim:**

To showcase our personal experience regarding reverse abdominoplasty and compare it to the state of the art.

**Methods:**

To identify indications, possible complications and outcomes, detailed surgical insights as well as graphical examples are provided. In addition, our personal experience from the last four years is showcased and compared with the literature using PubMed and Cochrane Library databases with Reverse AND Abdominoplasty as search strings.

**Results:**

All the 12 patients operated in our facilities between 2020 and 2024 had either a pre-existing submammary scar or a plan to undergo a contestual mammoplasty; at a mean of 25,1 weeks follow up, one major complication occurred.

**Discussion:**

There is a lack of publications on reverse abdominoplasty. Small case series are available in the literature, most of which focus on aesthetic indications. Only a few cases address the reconstructive implications of this surgical technique. In our experience, the concomitant desire or necessity of a mammoplasty and an already present inframammary scar favor the surgery. Careful recreation of a new inframammary sulcus must be considered to avoid unpleasant complications.

**Conclusions:**

Despite the poor literature supporting this technique, reverse abdominoplasty is a must-known procedure for successfully addressing thorny abdominal wall conditions and is characterized by consistent, replicable and safe outcomes.

## Introduction

Abdominoplasty and liposuction are two of the most common aesthetic procedures for reshaping the anterior trunk area. These techniques are effective and offer predictable results, especially when abundant soft tissue is placed beneath the umbilical area. Nevertheless, plastic surgeons may face complaints of patient dissatisfaction when an excess of soft tissue in the supraumbilical area is noted. This complication usually results from insufficient tissue shrinkage following liposuction or residual tissue redundancy, which can be worsened by plication of the muscular aponeurotic wall during abdominoplasty [1]. It is more visible when the patient is sitting and has the unfortunate characteristic of being exacerbated by clothes.

In these cases, surgeons are placed in the difficult position of deciding whether a revision of their previous surgery may bring an aesthetic improvement. We believe that reverse abdominoplasty, a relatively less-known procedure in which scars are placed in the inframammary folds and may or may not cross the midline, has the potential to solve the problem with safe and consistent outcomes in most cases.

This approach is especially useful if the desire for anterior wall contouring is associated with the intent of reshaping the breast *via* mastopexy, breast augmentation or breast reduction. In fact, the surgeon may remove the excessive skin in the abdominal area, leaving an inframammary scar identical to that of an inverted-T breast lift; moreover, the excess adipose tissue can be used to augment the breasts in some cases [2].

Reverse abdominoplasty can also be a valid alternative for patients who prefer to preserve the umbilical scar. In this case it can also be performed together with a mini-abdominoplasty to remove all the required excess tissue.

Through this work, we aim to provide an overview of reverse abdominoplasty with tips and tricks to maximize patient outcomes, with a concomitant review of the state of the art for this procedure.

## Materials and methods

A detailed step-by-step explanation of the indication, surgical procedure and possible complications will be provided. All the images and data shown below were obtained from our database of patients who underwent reverse abdominoplasty at our institutions between 2020 and 2024, who gave their consent to publish. To properly unfold the state of the art regarding this topic and to compare our outcomes, we reviewed the English literature using PubMed and Cochrane, with the search string ‘Reverse AND Abdominoplasty’. We emphasized the results of articles published in the last decade, as we believe them to be more relevant and up to date for the current discussion.

### Patient selection

**Figure 1.**
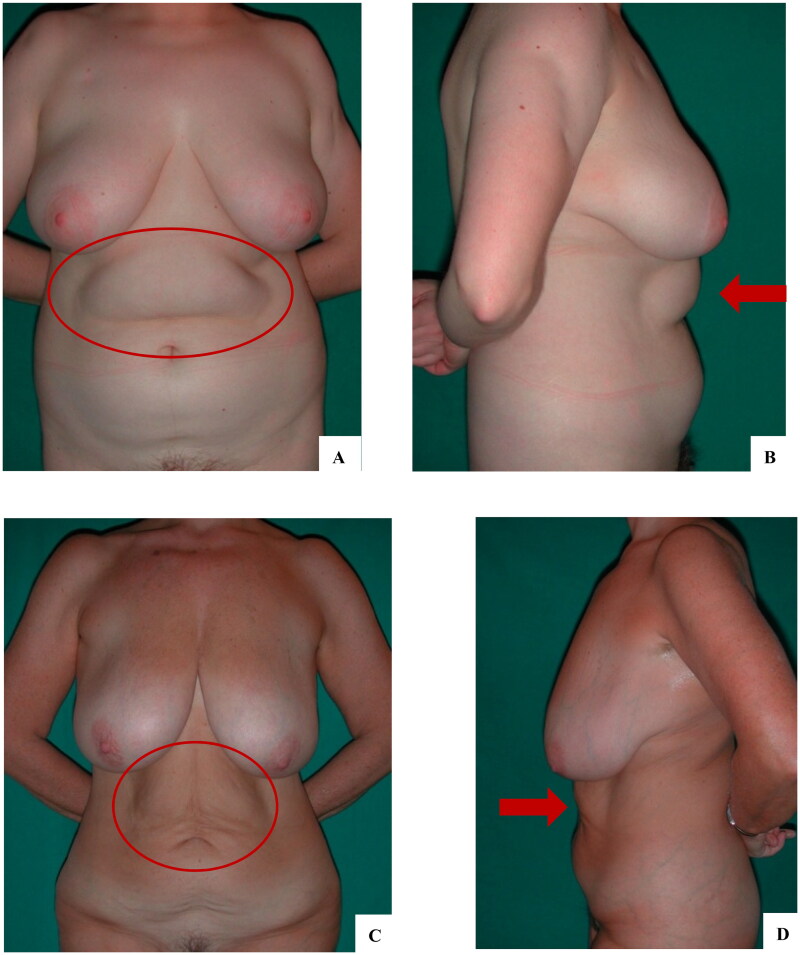
Patients eligible for reverse abdominoplasty have a soft tissue abundance in the upper abdominal region (circles and arrows). A, B: Diffuse adiposity in the abdomen favors reverse abdominoplasty with liposuction; the patient also underwent bilateral mastopexy. C, D: Skin excess in the supraumbilical region was associated with wide-based breasts that better hide the scar at the midline; for a better anterior body contouring, a mini-abdominoplasty was proposed, which the patient rejected.

Reverse abdominoplasty is indicated in patients with excess skin in the supraumbilical area after abdominoplasty and/or residual bulging of the abdomen after liposuction. The presence of previous scars in the inframammary area favor the indication, i.e. patients who underwent previous breast surgery, as well as patients who desire to leave the umbilical scar intact after the operation. The presence of a large base also favors the indication, as the scar will be better hidden underneath (Figure 1).

We found rejection of a scar crossing the midline and a history of keloids to be the only contraindications for this operation (Table 1).

**Table 1.** Indications and contraindications of reverse abdominoplasty.

Indication	Exclusion criteria
Epigastrial laxity	Rejection of midline scar
History of abdominoplasty/liposuction	History of cheloids
Inframammary scar	
Contestual mammoplasty indicated	
Desire not to operate the umbelical scar	
Large breast bases	

If the supraumbilical excess of skin is moderate, does not cross the midline, and if the patient has no muscle diastasis, reverse abdominoplasty without a midline scar can be proposed.

### Preoperative marking

**Figure 2.**
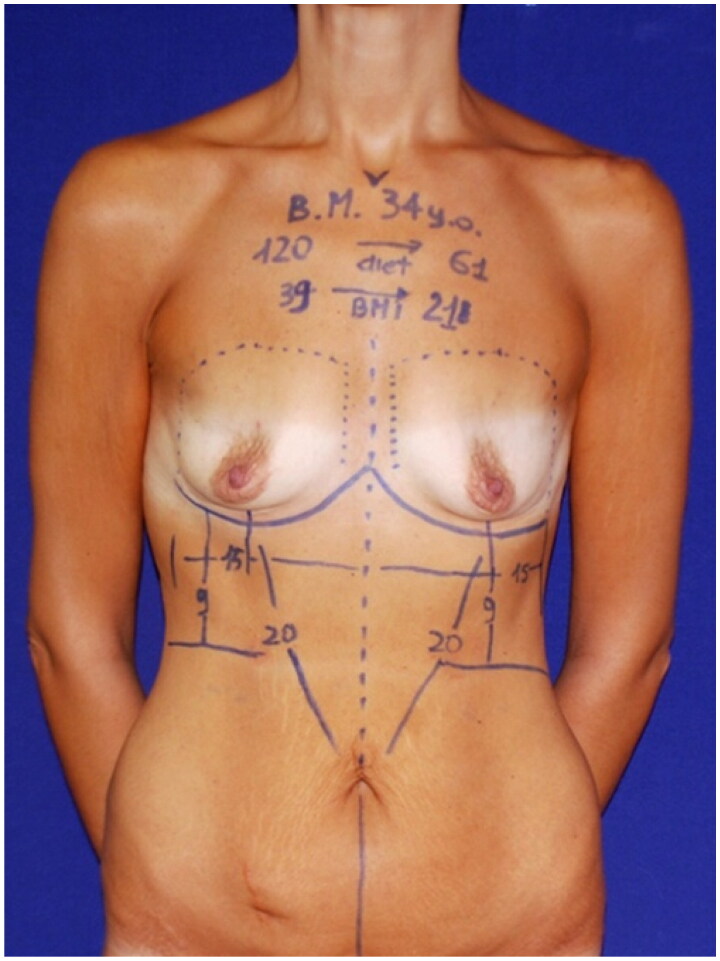
Preoperative marking in the orthostatic position of a patient undergoing reverse abdominoplasty and breast augmentation with implants. The new inframammary sulcus is drawn just beyond the original sulcus. The oblique lines indicate the area of flap dissection from the inframammary line to the umbilical scar. The vertical lines underline the proposed excised area.

Preoperative markings are made with the patient in the orthostatic position. First, the new inframammary sulcus and the midline are marked. Then, by pinching and pulling up the excess of skin, the lower border of the drawing is determined, similar to a classic abdominoplasty. At this point, the necessity of unifying the scars at the midline is evaluated (Figure 2).

**Figure 3.**
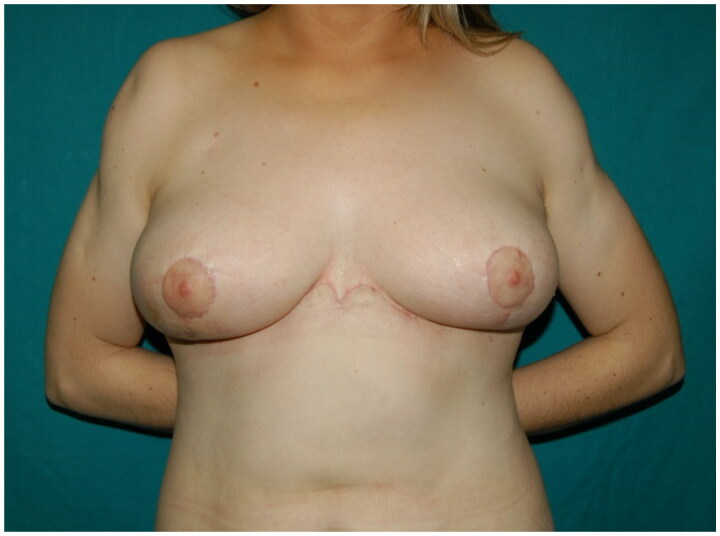
Detail of the midline scar. To avoid keloid formation in the sternal area, the junction of the inframammary scars at the midline should have an ‘M’ or ‘W’ shape.

If a scar crossing the midline is needed, it should have either a curvilinear or an ‘M/W’ shape to avoid the creation of symmastia and maintain the incision below the sternal region, notoriously inclined to form keloids (Figure 3).

### Surgical technique

**Figure 4.**
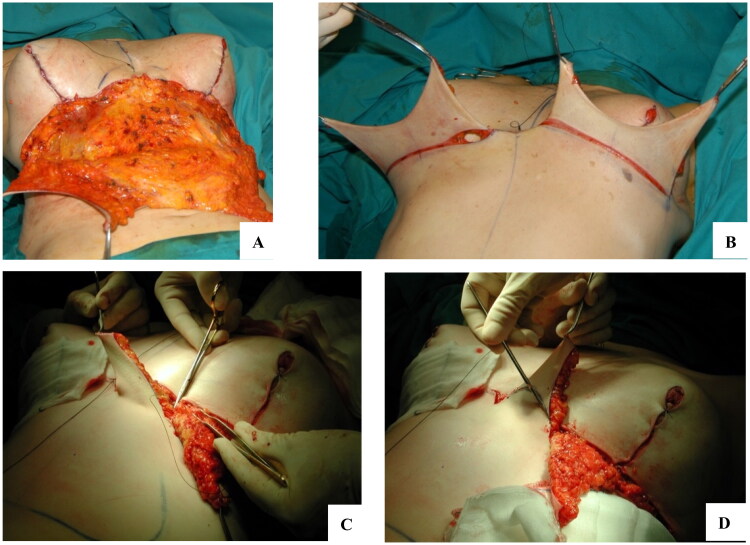
Intraoperative details of the procedure. A: Dissection of the flap above the aponeurotic plane. B: the flap is then pulled upward and split in half to estimate the excess of skin to be resected. C: Fixation of the new inframammary sulcus with deep sutures anchored to the periosteum of the ribs. D: to perform concomitant autologous breast augmentation, the flaps are split in half once again, deepithelialized and buried underneath the mammary gland.

With the patient positioned supine on the operating table, infiltration of the area with a saline solution enriched with adrenaline is performed. If the exceeding skin is supported by an excess of adipose tissue, liposuction of the area is advised. As in standard abdominoplasty, we avoid extensive liposuction of area 8 in order to preserve flap vascularization. Then, an incision in the upper part of the drawing is made, and dissection continued until the rectus abdominis fascia is reached. Then, dissection continues caudally until the umbilical scar is observed (Figure 4(A)). If the scar crosses the midline, a triangular area with the base at the inframammary line and the apex at the umbilicus is dissected; if no unification at the midline is needed, dissection is performed as two oblique tunnels from the inframammary sulcus of each breast that will be joined together approximately halfway between the xiphoid process and the umbilicus.

Then, the exceeding skin flap is pulled upward and excised as desired: using two Kocher clamps tractioned upward and slightly laterally, the surgeon marks the excess and sequentially removes it (Figure 4(B)). We advise moderate tension to avoid wound dehiscence, especially in post-bariatric patients. If necessary, plication of the recti is performed and, after meticulous revision of hemostasis, the flap tension is further controlled by anchoring it to the Scarpa’s fascia. We believe that six or seven knots disposed in the area of dissection of 2/0 braided absorbable sutures are adequate for most patients. Two drains are placed at the lateral aspect of the incision, and the skin is closed by three levels of sutures to ensure minimum tension at the epidermal layer. We use post-operative taping (for at least two weeks) and contenitive bra (for at least 6 weeks). Drains are usually removed on the first or second day after surgery.

### Adjunctive procedures

The additional procedures normally involve the breasts, with mastopexy, breast reduction mammaplasty or breast augmentation mammaplasty. The latter can be performed with an implant or with autologous tissue from the abdominal flap that, instead of being excised, will be de-epithelized, split in half at the midline and inset beneath the mammary gland in a pocket prepared in each breast (Figure 4(D)). Especially in this scenario, care must be taken in deciding the right amount of tension to avoid an excessively stretched flap that would result in liponecrosis of the new mammal tissue.

Regardless of a cincomitant breast surgery, fixation of a new inframammary sulcus must be assured with upmost care. A deep flap fixation to the perichondrium/periosteum of the ribs or to the pectoralis major muscle with a 0 or 1/0 braided absorbable suture is usually sufficient (Figure 4(C)). Improper fixation of the submammary sulcus can resolve in asymmetries or implant migration, which normally require reintervention.

## Results

Between 2020 and 2024, 12 patients underwent reverse abdominoplasty at our institution, which amount of less than 5% of all the abdominal wall operations in the same time span. Table 2 summarizes patient characteristics and follow up. In our cohort of females with a mean age of 50,3 years and a mean Body Mass Index of 24,4 kg/m^2^, all patients had either preexisting submammary scar or underwent contextual mammoplasty, with a 50% of patients who had both. At a mean 25,1 weeks of follow up one minor complication (small wound healing delay) and one major complication (sulcus asymmetry) were noted.

**Table 2.** Patient demographics, operative procedures, and follow-up.

Patient	Sex	Age (years)	BMI (operation time)	Previous abdominal operation	Preexisting submammary scars	Additional mammoplasty	Complication	Follow up (weeks)
1	F	66	23,2	A	yes	yes (AM)	none	4
2	F	55	24,8	none	no	yes (M)	sulcus asimmetry	24
3	F	33	26,5	A, L	yes	yes (AM)	none	33
4	F	77	22,7	none	yes	no	none	27
5	F	37	21,5	A	yes	yes (AM)	none	16
6	F	43	22,7	A	no	yes (AM)	none	36
7	F	48	25,2	none	no	yes (AM)	none	18
8	F	62	27,1	A	no	yes (AM)	none	48
9	F	45	21,3	A	yes	yes (AM)	none	26
10	F	39	27,4	A, L	yes	yes (M)	none	32
11	F	61	25,4	A, L	yes	yes (BR)	healing delay	8
12	F	37	25,3	A, L	yes	no	none	29
Mean		50, 3	24,4					25,1

BMI: body mass index; F: female; A: abdominoplasty; L: liposuction; AM: augmentation mammoplasty; M: mastopexy; BR: breast reduction.

## Discussion

Reverse abdominoplasty is a lesser-known surgical procedure used to address anterior wall deformities. It shares with conventional abdominoplasty the necessity to resect an excess of soft tissue from the abdominal wall, the plane of dissection, and the general principles regarding the tension of the flap and the level of the sutures. The site of incision and the relationship with the umbilical scar (generally not dissected) are the main differences.

The first known publication was released by Rebello and Franco in 1972 in the Spanish literature and then in 1977 in the English literature [3]. In this article, the authors described the procedure, indicated for ‘bulgy epigastric region’, in combination with a reduction mammaplasty and, in some patients, even with conventional abdominoplasty. In the English literature, the procedure was popularized by Baroudi et al. in 1979 [4]. They also used this technique with simultaneous breast reduction. The procedure was then relatively left in the background, with most of the scientific interest drawn towards either classical abdominoplasty or liposuction. More recently, some case series were published: Halbesma et al. [5] and Pacifico et al. [1] described a series of 7 and 14 patients, respectively. Mauro F. Deos et al. proposed an alternative anchoring of the flap to the overlying abdominal skin, which was called tensioned reverse abdominoplasty. The possibility of combining reverse abdominoplasty with an autologous breast augmentation was first described by Hurwitz and Agha-Mohammadi [6] as the ‘spiral flap’, and subsequently, by Zienowicz et al. who proposed augmentation *via* reverse abdominoplasty (AMBRA) [7].

**Figure 5.**
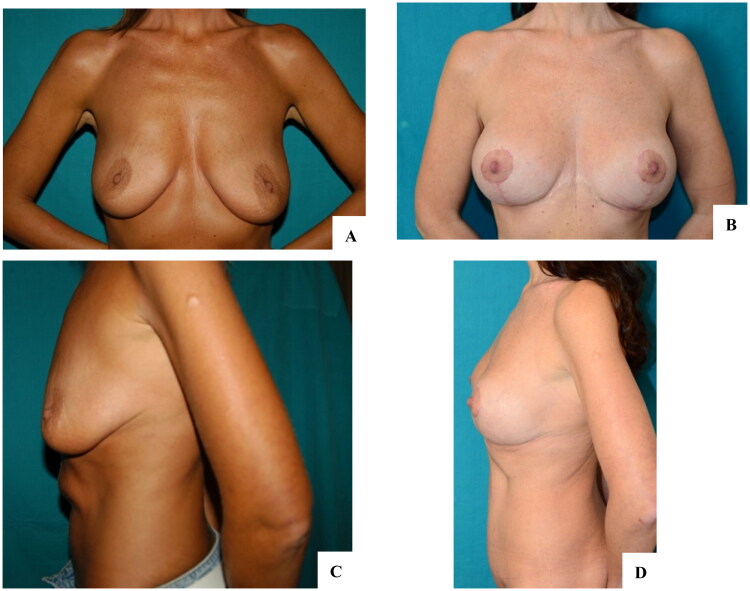
Case report of reverse abdominoplasty combined with breast lift with implants. A, C: Preoperative images showing mammary ptosis associated with excess of skin in the epigastric region. B, D: One-year follow-up reports satisfactory outcomes and absence of wall deformities.

**Figure 6.**
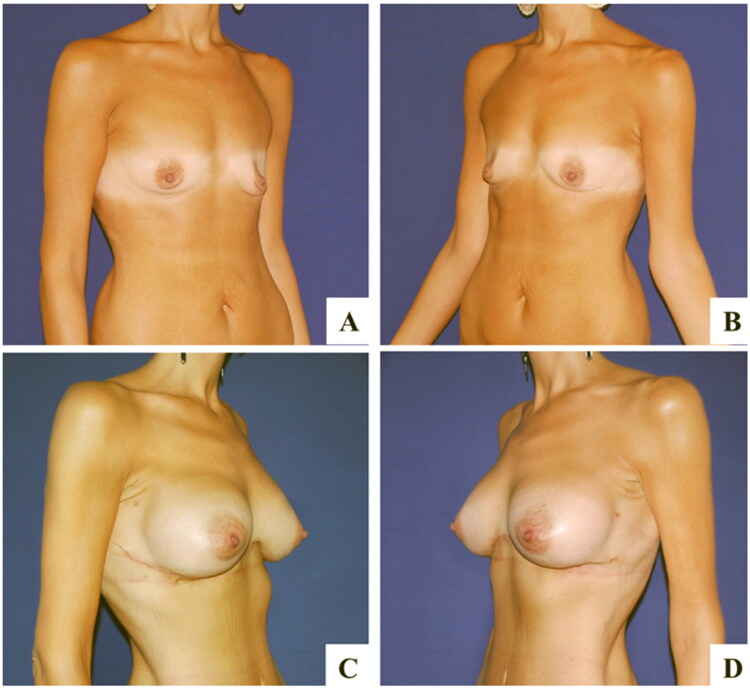
Case report of reverse abdominoplasty with breast augmentation. A, B: Preoperative findings of severe mammary hypotrophy after bariatric surgery. The patient also complained of a light excess of upper abdominal skin, which was exacerbated while sitting. C, D: Three-month follow-up showing a flat abdomen and correction of the breast deformity with implants.

In our experience, we propose reverse abdominoplasty either as a primary surgery, especially in presence of a wide-based breast to better hide the scars, or in patients who request a combined procedure involving mammaplasty (Figure 5 and 6). We found the latter to be more likely to undergo surgery because they tend to complain less about the incision. Similarly, a patient with a previous submammary scar generally accepts a scar crossing the midline. We also propose reverse abdominoplasty combined with mini-abdominoplasty in patients eligible for a fleur-de-lis abdominoplasty who reject a vertical incision, either as a two-stage surgical program or single-stage surgery, as proposed by Xiao Yang et al. [8]

**Figure 7.**
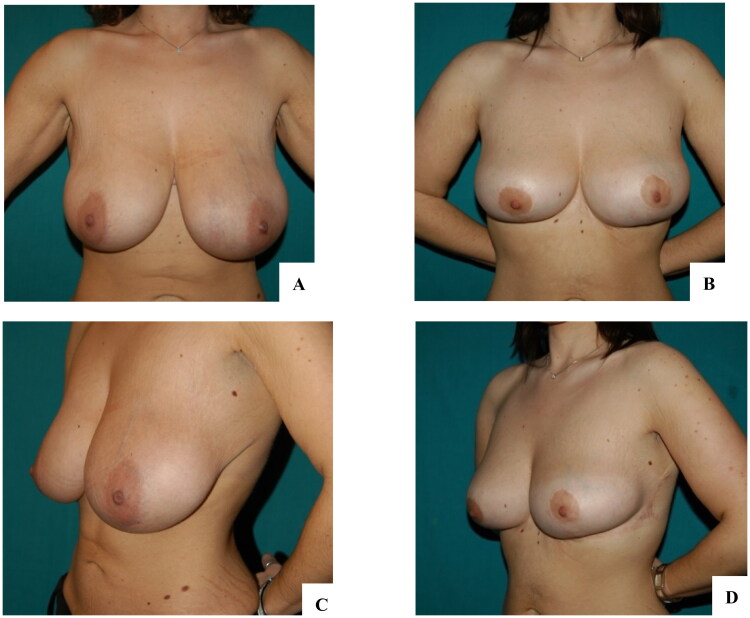
Complication after reverse abdominoplasty with breast reduction. After surgery, improper fixation of the right inframammary sulcus resulted in cranial rise of it with consequent unaesthetic mammary asymmetry. The patient underwent revision surgery to fix the sulcus in the proper position.

75% of our patients had a history of previous abdominoplasty or liposuction, which did not properly resolve the excess upper abdomen skin. Our findings are in line with the work of Pacifico et al. who reported similar indications [1]. As underlined in their work, special care must be taken in recreating the new inframammary sulcus with deep superficial suspension. The only major complication we faced was in a case of reverse abdominoplasty combined with breast reduction: improper fixation of one inframammary sulcus resulted in a poor aesthetic outcome that required a revision surgery (Figure 7). Other minor complications are usually patient- dependent: in the post-bariatric population, wound healing delays and seroma formations are usually the most frequent.

This surgical procedure also has reconstructive indications, especially for patients suffering abdominal skin laxity where previous abdominal scars are present. Case reports of reverse abdominoplasty as a salvage procedure after infected mammary implant and for treating a patient with previous nephrectomy and transverse muscular hernia scars have been published [9,10].

## Conclusion

Management of supra-umbilical skin laxity can be challenging for most physicians. Despite not having a rich literature support, reverse abdominoplasty is a must-known procedure for every plastic surgeon that should be considered in case of redundant upper abdominal wall tissue after previous abdominal wall contouring surgeries. This approach ensures consistent and safe outcomes when diligently performed. Perks are exacerbated and pitfalls minimized if a concomitant breast procedure is to be proposed, even though this technique can also be offered as a primary surgical act in the right-suited patient.
